# A rare case of miliary blastomycosis

**DOI:** 10.1016/j.rmcr.2025.102203

**Published:** 2025-04-03

**Authors:** Laura E. Ramirez, Alexander K. Foyt, Noureldien Darwish, Llewellyn Foulke, Christian Kostowniak, Amit Chopra

**Affiliations:** a Albany Medical College, Albany, NY, USA; b Department of Pathology, Albany Medical College, Albany, NY, USA; c Department of Medicine, Albany Medical College, Albany, NY, USA; d Department of Medicine, Division of Pulmonary and Critical Care Medicine, Albany Medical College, Albany, NY, USA

## Abstract

Blastomycosis is a fungal infection caused by the fungi *Blastomyces dermatitidis.* Presentation from this infection is variable, ranging from asymptomatic cases to disseminated infection. The Capital District of New York is not traditionally considered an endemic area; however, the increasing number of reported cases suggests it may be an emerging endemic region for Blastomycosis. Thus, we present a case of pulmonary blastomycosis with a rare diffuse miliary pattern in an immunocompetent patient from CDNY. This case highlights that pulmonary blastomycosis can lead to severe disseminated infections, even in immunocompetent individuals, emphasizing the importance of heightened clinical suspicion and timely diagnosis.

## Highlights

• Blastomycosis is an emerging fungal infection in the region the Capital District of New York.
• Blastomycosis infection can cause life threatening complications in immunocompetent patients.
• Diagnosis of blastomycosis requires a high index of clinical suspicion in pulmonary infections with extrapulmonary lesions.
• Itraconazole is the preferred treatment for mild infections. Amphotericin B is used for severe or disseminated cases.

## 1 Introduction

Blastomycosis is considered endemic to North America, particularly in regions near the Great Lakes [[1], [2], [3]]. However, an increasing number of cases have been reported in the Capital District of New York (CDNY), suggesting the possibility that CDNY may be an emerging endemic area for blastomycosis [4]. Due to the nonspecific symptoms of the disease and the fact that CDNY is not considered an endemic area, Blastomycosis infections are often misdiagnosed, leading to delays in patient care. In this report, we present a case of an immunocompetent patient from the CDNY diagnosed with pulmonary blastomycosis with a diffuse miliary pattern.

## 2 Case presentation

A 36-year-old male with no past medical history presented to the emergency room for progressive shortness of breath, nonproductive cough, night sweats, and fever for three days. He denied any chest pain, nausea, vomiting, hemoptysis, or recent illness. His past medical history is only significant for tobacco abuse. He is from Puerto Rico and moved to the Capital District of New York six years ago. The patient reported no recent travel or known exposure to tuberculosis.

On presentation, the patient appeared to be in mild respiratory distress. His vital signs were notable for a temperature of 100.4 °F, blood pressure of 128/81 mmHg, heart rate of 110 beats per minute, respiratory rate of 27 breaths per minute, and oxygen saturation of 93 % while receiving oxygen via high-flow nasal cannula at 2 L/min. Lung auscultation revealed decreased bilateral breath sounds, while the remainder of the physical examination was unremarkable.

Initial laboratory results showed significant leukocytosis with a white blood cell count of 21.7 × 10^3^/μL, thrombocytosis with a platelet count of 534 × 10^3^/μL, an elevated sedimentation rate of 112 mm/hr, a markedly high C-reactive protein level of 293.8 mg/L, and an increased D-dimer level of 1.74 mg/L FEU.

**Fig. 1:**
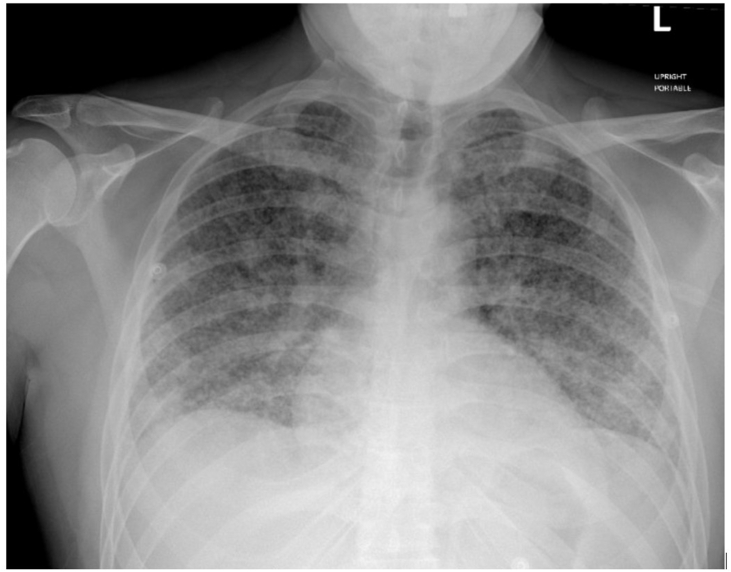
Chest radiograph showing diffuse miliary pattern of micronodularity in both lungs.

**Fig. 2:**
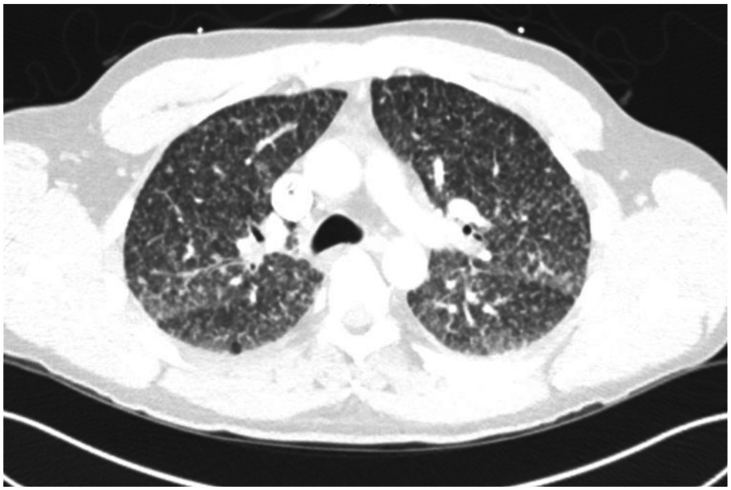
Chest CT scan showing bilateral diffuse micronodularity scattered throughout the lungs with a diffuse miliary pattern.

Chest radiography revealed diffuse bilateral micronodules (Fig. 1). Computed tomography (CT) scan was done to further evaluate chest radiographic findings. CT scan further demonstrated widespread bilateral micronodularity throughout the lungs, consistent with a miliary pattern (Fig. 2).

Empiric antibiotic therapy was initiated in the emergency department with intravenous ceftriaxone (2 g) and azithromycin (500 mg). The patient was admitted to the medicine ward for further management of atypical pneumonia. He required oxygen supplementation via nasal cannula at 2 L/min. Blood and respiratory cultures and sputum for acid fast bacilli (AFB) stain were nonrevealing. Immunodiffusion Blastomyces antibody test and serum cryptococcal antigen test were also negative.

**Fig. 3:**
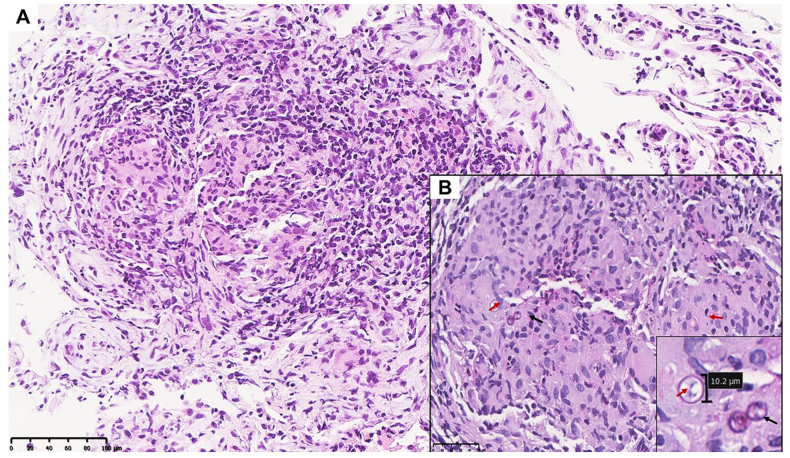
Transbronchial biopsy with blastomycosis. **A**. Low power image (H&E stain, 200x). **B.** Inserted higher power image (PAS stain, 400x).

Diagnostic bronchoscopy with bronchoalveolar lavage and transbronchial biopsies was performed to determine the cause of the nonspecific symptoms and diagnostic image findings. Bronchoscopy demonstrated normal airway anatomy and minimal secretions. However, on the 8th day of hospitalization, transbronchial biopsy results confirmed a fungal infection caused by *Blastomyces dermatitidis* (Fig. 3).

Following confirmation of the diagnosis, Amphotericin B (5 mg/kg IV every 24 hours) was initiated for 14 days. Throughout the course of therapy, his respiratory status improved, oxygen support was weaned, leukocytosis resolved, and his overall clinical condition significantly stabilized. He remained hospitalized for the entire duration of Amphotericin B treatment and was discharged after a 21-day hospital stay. Following discharge, he was closely monitored by the infectious disease team and started on itraconazole 200 mg twice daily, with a treatment duration of 6–14 months.

## 3 Discussion

Blastomycosis dermatitis is a thermally dimorphic fungus that forms a filamentous mold in the environment and as a yeast in the human body. The yeast-like cells demonstrate thick walls, broad-based budding, multiple nuclei, and a typical diameter range of 8–15 μm [1]. The Mississippi and Ohio River Valley regions of the United States and four Canadian provinces (Quebec, Ontario, Manitoba, and Saskatchewan) are highly endemic areas [2]. The Capital District of New York is not considered an endemic area. However, recent data shows that Blastomycosis is an emerging disease in the region, especially in areas near the Mohawk River valley [4]. Transmission occurs through inhalation of the conidia produced by the mold, which travels to the lungs, where it adopts the yeast-like form. Infection often occurs sporadically from environmental exposure. Outbreaks are usually caused by soil disruption due to construction, excavation work, and recreational activities such as hunting, fishing, and camping [1,2].

The clinical presentation of Blastomycosis is highly variable, ranging from asymptomatic to acute respiratory distress syndrome (ARDS). Due to the significant heterogeneity and nonspecific presentation, blastomycosis is often mistaken for other conditions such as community-acquired pneumonia, tuberculosis, and lung neoplasm [1]. Symptom onset may begin 3 weeks to 3.5 months after initial spore inhalation. Symptomatic infection typically presents with cough, fever, fatigue, chest pain, and dyspnea. However, approximately 50 % of people who become infected have either subclinical or asymptomatic presentation [1,2].

Because Blastomycosis enters the body through the lungs, pulmonary infection is reported in most documented cases. Extrapulmonary infection can occur in approximately 25 %–40 % of patients who develop symptoms [5]. Blastomycosis can disseminate to any organ; most commonly, it disseminates to the skin, bones, and genitourinary tract. Most patients with documented Blastomycosis are immunocompetent [6]. Nevertheless, immunocompromised patients, such as those infected with HIV and organ transplant recipients, are at higher risk of dissemination and may develop more serious complications [1,2].

Imaging findings of pulmonary blastomycosis are nonspecific, making the diagnosis challenging. Common radiographic X-ray findings include air-space consolidation with an alveolar pattern, which is one of the primary manifestations seen in chest radiographs, occurring in approximately 76 % of patients. The next most common radiographic manifestation seen in X-rays is mass-like infiltrations. Pleural effusions and lymphadenopathy occur in more than 20 % of cases [[7], [8], [9]].

The predominant computed tomography (CT) findings seen in most patients are pulmonary nodules, air bronchograms, and consolidation. Approximately one-third of patients present with enlarged lymph nodes, and pleural fluid is seen in one-quarter of cases [7]. Other less common abnormalities sometimes appreciable in CT include miliary pattern and pulmonary scarring. Acute pulmonary infections are more likely to show patchy infiltrates compared to chronic infections, which are more likely to show mass-like lesions or nodules mimicking malignancy [2,5]. Blastomycoses commonly involve upper lobes in segmental, nonsegmental, or lobar distribution. Cavitations can be seen in one-third of the cases. However, miliary patterns have rarely been described [8,9].

Fungal culture is the gold standard method for making a definitive diagnosis of Blastomycosis. Bronchoscopy with Bronchoalveolar lavage is highly valuable in patients with pulmonary Blastomycosis, yielding positive results in 92 % of cases. Additionally, noninvasive approaches such as sputum, tracheal secretions, and gastric washing cultures are very effective, identifying the fungi in 86 % of samples [10]. Samples are incubated at temperatures of 25 °C–30 °C to aid growth, and it can take 5–14 days before they can be visualized. Microscopic visualization of the broad-based budding and doubly refractile cell wall can assist in making an early diagnosis [1]. Since Blastomycosis does not gram stain well, stained sputum or tissue samples using calcofluor white, periodic acid-Schiff, 10 % KOH, or Gomori methenamine can help visualize the characteristic yeast form under the microscope in nearly 80 % of cases. Various antibody tests, including immunodiffusion (ID) and complement fixation, are available but tend to be unreliable due to low specificity and sensitivity [11]. Serum and urine enzyme immunoassay testing are helpful diagnostic tools, with sensitivities of 56 %–82 % and 76 %–90 %, respectively. The effectiveness of these tests are highly dependent on the burden of infection, yielding more accurate results in more severe forms of the disease. Polymerase chain reaction (PCR) is also a diagnostic option showing high specificity [1,10,11].

Immunocompetent and immunocompromised patients with disseminated infections, and patients with moderate to severe pneumonia require antifungal treatment [11]. Amphotericin B (3–5 mg/kg) once per day for 1–2 weeks or until improvement is the initial treatment in cases of moderate to severe pulmonary infection, immunocompromised patients, and disseminated Blastomycosis. Once Amphotericin B is completed, it is recommended to transition to oral Itraconazole (200 mg) 3 times a day for 3 days and then twice a day for a period of 6–12 months. Oral itraconazole is recommended for 6–12 months in cases of mild to moderate infection. Immunocompromised patients might require lifelong suppressive treatment with oral Itraconazole (200 mg) daily in cases where immunosuppression has not resolved [12].

The mortality rate is highly dependent on the state of the disease. The overall mortality rate is approximately 6.6 %. In immunocompromised patients, the mortality rate is much higher, around 36 %. Blastomycosis-induced ARDS is associated with high mortality rates of 75 %, which can be even higher in immunocompromised patients [13].

## 4 Clinical course

The patient had Pulmonary Blastomycosis. He likely acquired the infection near his residency in Schenectady, NY, which is near the Mohawk River, a new emerging endemic area for Blastomycosis. The bronchoscopy confirmed the diagnosis of Blastomycoses, and the patient was started on IV Amphotericin B for 2 weeks. The patient drastically improved, and the treatment was followed with oral Itraconazole for 12 months.

## CRediT authorship contribution statement

**Laura E. Ramirez:** Writing – review & editing, Writing – original draft, Supervision, Resources, Project administration, Methodology, Investigation, Funding acquisition. **Alexander K. Foyt:** Writing – review & editing, Writing – original draft, Visualization, Methodology, Funding acquisition. **Noureldien Darwish:** Writing – review & editing, Visualization, Resources, Investigation, Data curation. **Llewellyn Foulke:** Writing – review & editing, Visualization, Resources, Data curation. **Christian Kostowniak:** Writing – review & editing, Resources, Investigation. **Amit Chopra:** Writing – review & editing, Writing – original draft, Visualization, Supervision, Resources, Methodology, Investigation, Funding acquisition, Formal analysis, Conceptualization.

## Declaration of competing interest

The authors declare that they have no known financial or personal conflicts of interest that could have influenced the work reported in this manuscript.
